# Impact of Intratumoral Expression Levels of Fluoropyrimidine-Metabolizing Enzymes on Treatment Outcomes of Adjuvant S-1 Therapy in Gastric Cancer

**DOI:** 10.1371/journal.pone.0120324

**Published:** 2015-03-20

**Authors:** Ji-Yeon Kim, Eun Shin, Jin Won Kim, Hye Seung Lee, Dae-Won Lee, Se-Hyun Kim, Jeong-Ok Lee, Yu Jung Kim, Jee Hyun Kim, Soo-Mee Bang, Sang-Hoon Ahn, Do Joong Park, Jong Seok Lee, Ju-Seog Lee, Hyung-Ho Kim, Keun-Wook Lee

**Affiliations:** 1 Department of Internal Medicine, Seoul National University College of Medicine, Seoul National University Bundang Hospital, Seongnam, Korea; 2 Department of Pathology, Seoul National University College of Medicine, Seoul National University Bundang Hospital, Seongnam, Korea; 3 Department of Surgery, Seoul National University College of Medicine, Seoul National University Bundang Hospital, Seongnam, Korea; 4 Department of Systems Biology, The University of Texas MD Anderson Cancer Center, Houston, Texas, United States of America; Duke Cancer Institute, UNITED STATES

## Abstract

We analyzed the expression levels of fluoropyrimidine-metabolizing enzymes (thymidylate synthase [TS], dihydropyrimidine dehydrogenase [DPD], thymidine phosphorylase [TP] and orotate phosphoribosyltransferase [OPRT]) to identify potential biomarkers related to treatment outcomes in gastric cancer (GC) patients receiving adjuvant S-1 chemotherapy. In this study, 184 patients who received curative gastrectomy (D2 lymph node dissection) and adjuvant S-1 were included. Immunohistochemistry and quantitative reverse transcription polymerase chain reaction were performed to measure the protein and mRNA levels of TS, DPD, TP, and OPRT in tumor tissue. In univariate analysis, low intratumoral DPD protein expression was related to poorer 5-year disease-free survival (DFS; 78% vs. 88%; P = 0.068). Low intratumoral DPD mRNA expression (1st [lowest] quartile) was also related to poorer DFS (69% vs. 90%; P < 0.001) compared to high intratumoral DPD expression (2nd to 4th quartiles). In multivariate analyses, low intratumoral DPD protein or mRNA expression was related to worse DFS (P < 0.05), irrespective of other clinical variables. TS, TP, and OPRT expression levels were not related to treatment outcomes. Severe non-hematologic toxicities (grade ≥ 3) had a trend towards more frequent development in patients with low intratumoral DPD mRNA expression (29% vs. 16%; P = 0.068). In conclusion, GC patients with high intratumoral DPD expression did not have inferior outcome following adjuvant S-1 therapy compared with those with low DPD expression. Instead, low intratumoral DPD expression was related to poor DFS.

## Introduction

Gastric cancer (GC) is the fifth most common cancer and the third leading cause of cancer death worldwide. About half of the cases occur in Eastern Asia [1]. Surgery is the treatment of choice in localized GC, and D2 dissection is considered the standard approach. Despite D2 dissection, recurrence is noted in more than 40% of patients with advanced stage GC after surgery alone [2, 3]. Recently, two prospective studies showed that adjuvant chemotherapy for resected GC is effective in reducing the recurrence rate [2, 3].

S-1 is an oral agent containing tegafur (a prodrug of fluorouracil), gimeracil (an inhibitor of dihydropyrimidine dehydrogenase [DPD]), and potassium oxonate (an inhibitor of 5-fluorouracil [5-FU] in the gastrointestinal tract) [4]. The Adjuvant Chemotherapy Trial of TS-1 for Gastric Cancer (ACTS-GC) revealed that adjuvant S-1 chemotherapy for 1 year reduces tumor recurrence in patients with curatively resected GC [3]; the relapse-free survival rate at 5 years was 65.4% in the adjuvant S-1 group and 53.1% in the surgery-only group (hazard ratio [HR], 0.653; 95% confidence interval [CI], 0.537 to 0.793). Based on those results, adjuvant S-1 chemotherapy is currently widely used for preventing GC relapse in East Asian countries [5–7].

Thymidylate synthase (TS), thymidine phosphorylase (TP), orotate phosphoribosyltransferase (OPRT), and DPD are related to fluoropyrimidine metabolism. Several studies suggested that the expression levels of these fluoropyrimidine-metabolizing enzymes have associations with survival outcomes in metastatic GC patients receiving palliative S-1-based chemotherapy [8–10]. However, those studies showed inconsistent results on the predictive value of these enzymes in S-1-based palliative chemotherapy, which may be due to the small numbers of patients and differences in S-1-containing regimens used among studies. Up to the present, studies on the relationship between the expression levels of fluoropyrimidine-metabolizing enzymes and outcomes of adjuvant S-1 treatment in GC have been scarce. Therefore, we analyzed the protein and mRNA expression profiles of fluoropyrimidine-metabolizing enzymes (TS, DPD, TP and OPRT) to identify markers related to treatment outcomes of adjuvant S-1 chemotherapy in GC patients.

## Materials and Methods

### Patient population

Using a GC cancer patient cohort that was prospectively maintained at Seoul National University Bundang Hospital [5, 6], this study was retrospectively designed. In the prospective cohort, all patients underwent curative gastrectomy with D2 dissection and were treated with adjuvant S-1 chemotherapy. The patients met the following eligible criteria: histologically confirmed gastroesophageal junction or gastric adenocarcinoma; pathologic stage II-III using the American Joint Committee on Cancer (AJCC, 7^th^ edition); Eastern Cooperative Oncology Group (ECOG) performance status (PS) 0–2; and adequate bone marrow, renal, and hepatic function. Two patients with stage IB and additional risk factors (i.e., N2 lymph node metastasis by Japanese staging classification [11]) were also included.

Patients who underwent gastrectomy between November 2006 and September 2010 were enrolled (N = 184). For this study, written informed consents were received from patients for using archived tumor tissues and clinical data. The Institutional Review Board of Seoul National University Bundang Hospital approved this study (IRB number: B-1205/154–006).

### Treatment and toxicity assessment

S-1 was given orally for 4 weeks, followed by 2 weeks of rest. The duration of S-1 treatment was planned to be 1 year if there was no evidence of tumor recurrence, unacceptable adverse events, or patient refusal. The initial dosage and modification of S-1 dosage during treatment were determined as in previous reports [5, 6]. Toxicity was graded according to the National Cancer Institute Common Toxicity Criteria (version 3.0).

The dose intensity (DI) was defined as the ratio of the total S-1 dose per square meter of the patient, divided by the total S-1 treatment duration. The relative dose intensity (RDI) was calculated by dividing the received DI by the planned DI.

### Immunohistochemistry (IHC)

Previously stained hematoxylin and eosin slides were reviewed, and one representative formalin-fixed paraffin-embedded (FFPE) archival block was selected. Tissue array blocks were prepared as described in our previous study (SuperBiochips Laboratories, Seoul, Korea) [8]. The following primary antibodies were used: anti-human TS (clone TS106; mouse monoclonal; Thermo scientific; 1:70 dilution), anti-human OPRT (clone 2F5; mouse monoclonal; Abnova; 1:1000 dilution), anti-human TP (clone P-GF. 44C; mouse monoclonal; Thermo scientific; 1:500 dilution), and anti-human DPD (clone ERP8811; rabbit monoclonal; Abcam; 1:500 dilution). Antibody binding was detected using the avidin-biotin-peroxidase complex (Universal Elite ABC kit PK-6200; Vectastain, Burlingame, CA, USA) for 10 min and diaminobenzidine tetrahydrochloride solution (Kit HK 153–5K; Biogenex, San Ramon, CA, USA). The sections were counterstained with 0.1% hematoxylin, dehydrated, and mounted. The staining was examined by two independent investigators blinded to the clinical outcome. Staining intensities were semi-quantitatively measured at a 200× magnification and categorized as negative (score = 0), weak (score = 1), moderate (score = 2), or strong (score = 3). The percentage of immunoreactive cells was also assessed. The IHC score was calculated as follows: the score of staining intensity was multiplied by the percentage of stained area of the tumor sample.

### Quantitative reverse transcription polymerase chain reaction (qRT-PCR)

The mRNA expression levels of genes were quantified by qRT-PCR. RNA (500 ng) from FFPE tissues was subjected to cDNA synthesis using the amfiRivert Platinum cDNA Synthesis Master Mix (GenDEPOT). Seven genes (four fluoropyrimidine pathway genes and three reference genes) were pre-amplified at a final dilution of 0.05× original Taqman assay concentration (Applied Biosystems by Life Technologies). The thermo-cycling conditions were as follows: 1 cycle of 95°C (10 min), followed by 14 cycles of 95°C (15 s) and 60°C (4 min). Following target amplification, samples were diluted 1:5 with DNA suspension buffer, and then qPCR was carried out on Fluidigm 48.48 Dynamic Arrays using the BioMark HD system according to the manufacturer’s protocol. Samples were run in triplicate. Three reference genes (ACTB, GAPDH, and FTL) were used for normalization of gene expression data. Reference genes were selected based on their low expression variability in our previous microarray data (data not shown). For normalization of qRT-PCR data, mean cycle threshold (Ct) values were converted to relative expression values (-ΔCt) by subtracting the mean of the reference genes, where each unit reflects a 2-fold increase in expression. The used probes were as follows: TS (Hs00426586_m1), OPRT (Hs00923517_m1), TP (Hs01034319_g1), DPD (Hs00559279_m1), ACTB (Hs01060665_g1), GAPDH (Hs02758991_g1) and FTL (Hs00830226_gH); more detailed information is available at http://www.appliedbiosystems.com (Applied Biosystems by Life Technologies).

### Statistical analysis

Differences in clinical characteristics were compared using χ2-tests, *t*-tests, or the Mann–Whitney *U* test. χ2-tests were used in comparing the frequency distributions of S-1-related toxicities between different groups. Disease-free survival (DFS) was calculated from the date of surgery to the time of first recurrence or death from any cause. Overall survival (OS) was defined as the interval from surgery to death from any cause. The Kaplan–Meier method was used to analyze DFS or OS. Univariate and multivariate analyses on DFS or OS were conducted using the log-rank tests and Cox proportional hazards regression tests, respectively. Two-sided P values of < 0.05 were considered significant. All data analyses were conducted using IBM SPSS Statistics 21 for Windows (IBM Corp., Armonk, NY, USA).

## Results

### Patient characteristics and delivery of S-1 chemotherapy

**Fig 1:**
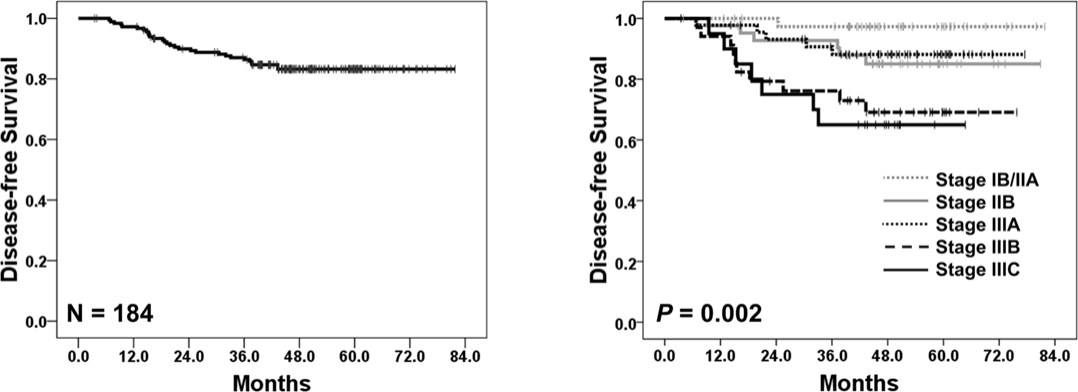
Disease-free survival (A) in all patients (N = 184) and (B) according to stages.

The baseline characteristics of the 184 patients are shown in Table 1. The median age was 57 years (range, 30–79 years). Laparoscopic gastrectomy was performed in 50% of patients, and the proportion of patients with stage III disease was 55%. The median follow-up duration was 47.0 months (range, 3.5–81.8 months). During follow-up, tumor recurrence and death were confirmed in 29 (16%) and 22 (12%) cases, respectively. Five-year DFS and OS rates in all patients were 83% (Fig. 1(A)) and 87%, respectively. As follow-up duration was not sufficient for further OS analysis, we performed survival analysis only for DFS. DFS outcomes according to clinical variables are presented in Table 1, Fig. 1(B), and S1 Fig.

**Table 1:** Patient characteristics and disease-free survival according to clinical parameters.

	No.	5-year DFS (%)	P-value
**Sex**			0.585
Male	110	84.6%	-
Female	74	81.3%	-
**Age** (Median 57 years; Range 30–79 years)			0.003
< 60 years	102	85.7%	-
60–69 years	48	90.7%	-
≥ 70 years	34	64.7%	-
**ECOG PS**			0.515
0	66	85.7%	-
1–2^1^	118	81.8%	-
**Comorbidities**			0.218
CMI = 0	149	84.9%	-
CMI ≥ 1	35	76.5%	-
**Gastrectomy extent**			0.753
Total gastrectomy	68	84.0%	-
Partial gastrectomy^2^	116	82.9%	-
**Operation method**			0.082
Laparoscopic surgery	92	87.6%	-
Open surgery	92	78.9%	-
**Lauren classification**			0.288
Diffuse	103	84.6%	-
Intestinal	64	79.0%	-
Indeterminate/Mixed	17	91.7%	-
**Tumor location (in stomach)**			0.523
Upper third	43	77.8%	-
Middle third	44	89.0%	-
Lower third	66	81.4%	-
≥Two thirds of stomach	31	86.8%	-
**Lymphatic invasion**			0.068
No	48	91.7%	-
Yes	136	81.3%	-
**Venous invasion**			0.045
No	160	86.1%	-
Yes	24	70.8%	-
**Perineural invasion**			0.539
No	58	86.0%	-
Yes	126	83.2%	-
**Stage** ^**3**^			0.002
IB/IIA^4^	40	97.3%	-
IIB	43	85.0%	-
IIIA	46	88.4%	-
IIIB	35	69.1%	-
IIIC	20	65.0%	-
**Stage** ^**3**^			0.012
IB/II^4^	83	90.8%	-
III	101	77.1%	-

Abbreviations: ECOG PS, Eastern Cooperative Oncology Group performance status; CMI, Charlson Comorbidity Index;

^1^ Among 118 patients, 8 had an ECOG PS of grade 2.

^2^ Among 116 patients, 2 underwent proximal gastrectomy and 114 underwent distal gastrectomy.

^3^ Tumor was staged according to the American Joint Committee on Cancer criteria (7^th^ edition).

^4^ Three patients had stage IB and the other patients had stage II.

Planned 1-year treatment with S-1 was completed in 139 patients (76%), and the mean number of delivered S-1 chemotherapy cycles was 7.4 (95% CI, 7.0–7.8). Mean and median RDIs during all S-1 therapy cycles were 77% (95% CI, 73–81) and 88% (range, 4–100), respectively. S-1 treatment was overall tolerable, and toxicity profiles are presented in S1 Table.

### Impact of expression levels of fluoropyrimidine-metabolizing enzymes on treatment outcome: Univariate analyses

**Fig 2:**
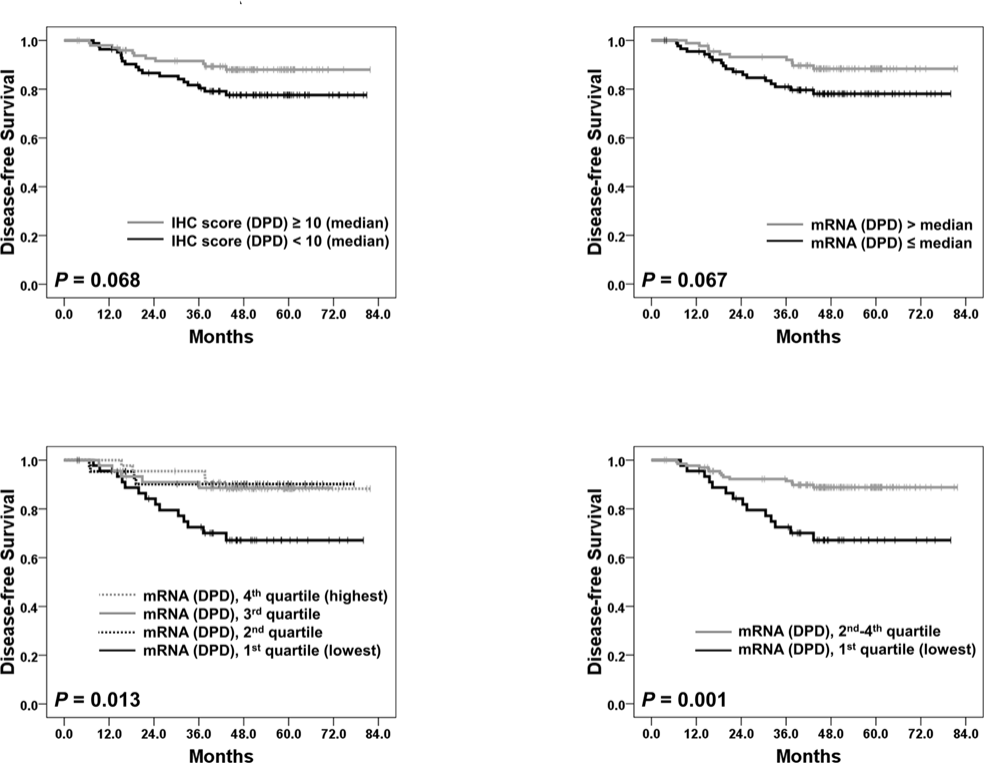
Disease-free survival according to the expression levels of DPD. (A) Disease-free survival according to the IHC sores of DPD (cut-off value; median). Disease-free survival curves according to intratumoral mRNA expression levels of DPD ((B) when the cut-off value is the median; (C) when patients are classified into quartiles; (D) when patients are classified into 2 groups [1^st^ quartile; the lowest quartile] vs. other quartiles [2^nd^, 3^rd^ and 4^th^ quartiles]). Abbreviations: IHC, immunohistochemistry; DPD, dihydropyrimidine dehydrogenase.

Of the 184 patients, IHC was successful in 183 patients. We set the median value of IHC scores for each protein (TS, OPRT, TP, and DPD) as the cut-off value to divide patients into two groups. While the expression levels of four proteins were not significantly related to DFS (Table 2), low DPD expression level (IHC score < 10 [cut-off value; median]) showed a trend towards worse 5-year DFS (78% vs. 88%; P = 0.068; Fig. 2(A)). In addition, patients with higher TP expression (IHC score > 0 [cut-off value; median]) had a tendency to have shorter DFS duration than those with no TP expression (78% vs. 87%; P = 0.094). The expression levels of TS and OPRT had no association with DFS outcomes (P = 0.914 and 0.109, respectively).

**Table 2:** Disease-free survival according to intratumoral protein or mRNA expression levels of fluoropyrimidine-metabolizing enzymes (univariate analyses).

Clinical variables	Cut-off value	Patient No.	5-year DFS (%)	P-value
**IHC score (N = 183)** ^**1**^				
TS (cut-off, 140 [median]; range, 0–300)	≤ 140	92	83.2%	0.914
	> 140	91	83.2%	
OPRT (cut-off, 3 [median]; range, 0–300)	≤ 3	94	87.9%	0.109
	> 3	89	78.3%	
TP (cut-off, 0 [median]; range, 0–300)	0	98	87.4%	0.094
	> 0	85	78.0%	
DPD (cut-off, 10 [median]; range 0–300)	< 10	83	77.6%	0.068
	≥ 10	100	88.0%	
**mRNA expression (N = 179)** ^**2**^				
TS (cut-off: median)	≤ median	90	87.2%	0.260
	> median	89	79.7%	
OPRT (cut-off: median)	≤ median	90	84.8%	0.726
	> median	89	81.9%	
TP (cut-off: median)	≤ median	90	86.0%	0.407
	> median	89	80.7%	
DPD (cut-off: median)	≤ median	90	78.1%	0.067
	> median	89	88.3%	
DPD (4 quartiles)	1^st^ quartile (lowest)	45	68.9%	0.013
	2^nd^ quartile	45	91.1%	
	3^rd^ quartile	45	88.9%	
	4^th^ quartile (highest)	44	88.6%	
DPD (cut-off; 1^st^ quartile)	1^st^ (lowest)	45	68.9%	0.001
	2–4^th^ quartiles	134	89.6%	

Abbreviations: IHC, immunohistochemistry; TS, thymidylate synthase; OPRT, orotate phosphoribosyltransferase; TP, thymidine phosphorylase; DPD, dihydropyrimidine dehydrogenase; qRT-PCR, quantitative reverse transcription polymerase chain reaction

^1^ Of 184 patients included in this study, IHC tests were successfully performed in 183 patients.

^2^ Of 184 patients, mRNA expression levels were successfully measured using qRT-PCR in 179 patients.

qRT-PCR tests were successfully conducted in 179 patients. As in IHC tests, a median value of mRNA expression levels of the individual genes was set as a cut-off value for groups. Among the four genes, only the DPD mRNA expression level showed a trend of having a relation to DFS outcomes; patients with lower mRNA expression of DPD had poorer DFS than those with higher DPD expression (P = 0.067; Fig. 2(B)). mRNA expression levels of the TS, OPRT, and TP genes were not related to DFS (Table 2). Next, we classified mRNA expression levels of DPD into quartiles to investigate whether a dose-response relationship existed between DPD gene expression levels and survival outcomes. Interestingly, only the patient group in the lowest DPD gene expression quartile showed inferior DFS to other quartiles, but no difference in DFS rates was observed between the 2^nd^, 3^rd^, and 4^th^ quartiles (Fig. 2(C)). Therefore, we classified patients into two groups using this cut-off value (1^st^ quartile [lowest DPD mRNA expression] vs. other quartiles [higher DPD mRNA expression including the 2^nd^, 3^rd^, and 4^th^ quartiles]) in the following analyses (Fig. 2(D); Table 2).

### Impact of expression levels of fluoropyrimidine-metabolizing enzymes on treatment outcome: Multivariate analyses

Among clinical variables, factors that showed P < 0.10 in univariate analyses for DFS (age [< 60 years vs. 60–69 years vs. ≥ 70 years; P = 0.003], surgical method [laparoscopic vs. open; P = 0.082], lymphatic invasion [no vs. yes; P = 0.068], venous invasion [no vs. yes; P = 0.045], and stage [IB/II vs. III; P = 0.012]; Table 1) were included in multivariate analyses. Among proteins or genes related to fluoropyrimidine metabolism, those with P < 0.10 in univariate analyses for DFS were also incorporated into multivariate analyses (Table 2). As shown in Table 3, low DPD protein expression level (IHC score < 10; HR, 2.32; 95% CI, 1.08–4.96; P = 0.030) and low DPD gene expression (1^st^ quartile [lowest quartile]; HR, 3.67; 95% CI, 1.67–8.03; P = 0.001) were related to poorer DFS, irrespective of other clinical variables. Older age (≥ 70 years) and higher stage were also independently associated with poor DFS.

**Table 3:** Impact of the expression levels of intratumoral DPD ((A) DPD protein expression and (B) DPD mRNA expression) on disease-free survival.

(A) IHC score (N = 183)	Hazard Ratio	95% Confidence Interval	P-value
**Age**			0.004
< 60 years	1.00		-
60–69 years	0.66	0.22–2.01	0.466
≥ 70 years	3.23	1.45–7.19	0.004
**Stage**			
IB/II	1.00	-	-
III	3.11	1.32–7.32	0.009
**DPD IHC score**			
≥ 10 (median)	1.00	-	-
< 10 (median)	2.32	1.08–4.96	0.030

**(B) mRNA expression (N = 179)**	**Hazard Ratio**	**95% Confidence Interval**	**P-value**
**Age**			0.022
< 60 years	1.00	-	-
60–69 years	0.52	0.17–1.63	0.262
≥ 70 years	2.31	1.00–5.33	0.049
**Stage**			
IB/II	1.00	-	-
III	3.31	1.39–7.90	0.007
**mRNA expression of DPD**			
2^nd^ to 4^th^ quartiles	1.00	-	-
1^st^ quartile (lowest quartile)	3.67	1.67–8.03	0.001

(A) Clinical variables that had P < 0.10 in univariate analyses on DFS (age [< 60 years vs. 60–69 years vs. ≥ 70 years], surgical method [laparoscopic vs. open], lymphatic invasion [no vs. yes], venous invasion [no vs. yes], stage [IB/II vs. III], DPD IHC score [≥ 10 vs. < 10] and TP IHC score [0 vs. > 0]) were included in this multivariate analysis using a Cox proportional hazards model. A backward stepwise conditional regression was used with P = 0.10 as the entry and P = 0.10 as the removal criteria. Abbreviations: IHC, immunohistochemistry; DPD, dihydropyrimidine dehydrogenase; TP, thymidine phosphorylase.

(B) Clinical variables that had P < 0.10 in univariate analyses on DFS (age [< 60 years vs. 60–69 years vs. ≥ 70 years), surgical method [laparoscopic vs. open], lymphatic invasion [no vs. yes], venous invasion [no vs. yes], stage [IB/II vs. III]) and mRNA expression levels of DPD gene were included in this multivariate analysis using a Cox proportional hazards model. A backward stepwise conditional regression was used with P = 0.10 as the entry and P = 0.10 as the removal criteria.

### Relationship between DPD expression level and tolerance to S-1 therapy

A positive correlation between DPD IHC scores and mRNA expression levels was observed (P = 0.022), although the ranges of IHC scores in the two groups (1^st^ quartile of DPD mRNA expression [DPD IHC score: median, 0; range, 0–200] vs. other quartiles [DPD IHC score: median, 10; range, 0–300]) overlapped in a considerable proportion of cases (S2 Fig).

No differences in S-1 RDI or toxicity frequency (hematologic or non-hematologic) from S-1 treatment were observed between patients with low (< 10) and high (≥ 10) DPD IHC scores [Table A in S2 Table]. However, compared to patients with higher intratumoral DPD mRNA levels (2^nd^ to 4^th^ quartiles), patients with the lowest DPD mRNA levels (1^st^ quartile) showed a tendency of developing severe non-hematologic toxicities (≥ grade 3) more frequently, although statistically insignificant (29% vs. 16%; P = 0.068); however, the incidence of ≥ grade 3 hematologic toxicities was not different between the two groups. In addition, although statistically insignificant, the proportion of patients who maintained S-1 RDI ≥ median value (87.8%) during adjuvant chemotherapy was lower in patients within the lowest quartile of DPD mRNA expression than in patients within other quartiles (40% vs. 54%; P = 0.111; Table B in S2 Table).

## Discussion

In this study, we explored the relationship between intratumoral expression levels of fluoropyrimidine-metabolizing enzymes and survival outcomes of adjuvant S-1 chemotherapy. High intratumoral DPD expression was not related to inferior survival outcomes; instead, low DPD expression was associated with unfavorable DFS in GC patients treated with adjuvant S-1therapy. The expression levels of other enzymes did not correlate to DFS. In our study, the expression levels of fluoropyrimidine-metabolizing enzymes were assessed by two methods, namely IHC and qRT-PCR.

DPD is the initial and rate-limiting enzyme in the catabolism of 5-FU. Several preclinical studies have shown that lower intratumoral level of DPD mRNA expression or activity is associated with better response to 5-FU and that higher DPD level in tumor cells is related to 5-FU resistance [12–14]. Similar findings have also been observed in patients with various solid tumors including GC [14–22]. The effectiveness of fluoropyrimidine chemotherapy in GC patients has been shown to be dependent on intratumoral DPD expression levels, either in palliative [22], adjuvant [19–21], or neoadjuvant [16–18] settings.

Among the three components comprising S-1, gimeracil is used as an inhibitor of DPD to maintain prolonged 5-FU concentrations in tumor tissues [23]. Although some studies have reported that the efficacy of S-1 is also affected by intratumoral DPD expression levels like other fluoropyrimidines [24, 25], the majority of studies have consistently shown that the effect of S-1 therapy is not influenced by intratumoral DPD expression levels, especially in GC [8–10, 19, 26–29]. Previous studies suggested that S-1 is more effective than 5-FU or other fluoropyrimidines in tumors with high DPD expression [19, 25, 28, 29]. The relationship between the efficacy of S-1-containing chemotherapy and the expression levels of fluoropyrimidine-metabolizing enzymes has been investigated in GC [8–10]; however, most of these studies included small patient numbers and were conducted on metastatic GC patients who received various S-1-containing regimens as palliative treatment. Therefore, previous results cannot be generalized to GC patients who undergo curative surgery and adjuvant S-1 chemotherapy.

In our study, overall treatment outcome of D2 gastrectomy followed by adjuvant S-1 chemotherapy was excellent; 5-year DFS rates of patients with stage IB/II and III were 91% and 77%, respectively (Table 1). Among the four analyzed enzymes (TS, DPD, OPRT, and TP), only the expression level of DPD was related to treatment outcome of S-1. DPD protein or mRNA overexpression did not correlate with inferior DFS in GC patients receiving adjuvant S-1 therapy; this observation is consistent with prior reports that S-1 is effective in tumors with high intratumoral DPD [19, 25, 29, 30].

Interestingly, in our patient cohort, low intratumoral DPD expression was related to worse DFS, compared to high DPD expression. This observation was unexpected and confusing because, until we finalized the data analyses of this study, there had been no study on the association between fluoropyrimidine-metabolizing enzymes and outcomes of adjuvant S-1 chemotherapy in GC patients. However, nearly at the same time when we reported the results of this study at an academic meeting [31], a study with the almost same design with ours was reported by the ACTS-GC investigators [32]. In that study, the ACTS-GC investigators analyzed the intratumoral expression of 4 genes (TS, DPD, OPRT, and TP) in patients enrolled in the ACTS-GC and investigated their possible roles as biomarkers for treatment outcomes. Like us, the ACTS-GC investigators also suggested that low DPD mRNA expression in tumors is related to unfavorable DFS; among GC patients who had received adjuvant S-1 chemotherapy (N = 401), patients with low intratumoral DPD mRNA expression had inferior 5-year DFS (60.8% vs. 70.8%; P = 0.039) and OS (66.8% vs. 78.0%; P = 0.015) than those with high DPD expression level [32]. Therefore, the studies from both the ACTS-GC group and our investigators reached the unexpectedly same result from independent patient cohorts.

As only patients who had received S-1 treatment were included in this study, it remains uncertain whether intratumoral DPD expression level is a predictive or prognostic marker in GC patients receiving adjuvant S-1 therapy. However, as the ACTS-GC had the control group of patients who had received surgery alone without adjuvant chemotherapy, the ACTS-GC investigators showed that the benefit of adjuvant S-1 therapy is mostly confined to GC patients with high intratumoral DPD mRNA expression [surgery followed by adjuvant S-1 therapy vs. surgery alone; HR on OS, 0.52 (95% CI; 0.38–0.72)]; in contrast, in patients with low intratumoral DPD expression, adjuvant S-1 therapy seemed to have less benefit [surgery followed by adjuvant S-1 therapy vs. surgery alone; HR on OS, 0.85 (95% CI; 0.56–1.28)] [32]. Therefore, considering the study results from both ACTS-GC investigators and us, we cautiously suggest that the intratumoral DPD mRNA expression might be a predictive biomarker for the efficacy of adjuvant S-1 chemotherapy in GC patients after D2 gastrectomy. However, our suggestion needs to be further investigated in future prospective trials.

If the intratumoral DPD expression level is a predictive biomarker of adjuvant S-1 chemotherapy in GC patients, the underlying reasons why low intratumoral DPD expression confers inferior DFS outcome on GC patients receiving adjuvant S-1 therapy will require further investigation. First, although the authors from the ACTS-GC group did not investigate the association between toxicity profiles or RDI of S-1 and the level of intratumoral DPD expression [32], we analyzed differences in toxicity frequencies and RDI of S-1 according to the intratumoral DPD expression levels. We could not find any differences in toxicities and RDI between DPD-high and DPD-low patients by IHC score. However, although statistically insignificant, patients with the lowest intratumoral DPD mRNA levels (1^st^ quartile) showed a trend towards more non-hematologic toxicities of ≥ grade 3 (29% vs. 16%; P = 0.068) and seemed to have lower tolerance to S-1 than did patients with higher DPD mRNA levels (S2 Table). In a previous study [5], we reported that RDI of S-1 is related to the DFS of GC patients receiving adjuvant S-1 therapy and that the most frequent cause of S-1 dose reduction was enterocolitis, one of the most common non-hematologic toxicities developed during adjuvant S-1 therapy. Although whether DPD expression levels correlate between tumor cells and normal host cells remains unknown, Bertino et al. [15] reported that low intratumoral DPD expression may be associated with increased toxicity from capecitabine in lung cancer patients. Cui et al. [33] reported that serum DPD expression level is associated with the development of toxicity from S-1-based chemotherapy in metastatic GC patients. Taken together, we hypothesize that intratumoral DPD expression levels may reflect overall DPD levels in normal host tissues, indicating that low intratumoral DPD expression may be related to more frequent development of S-1 toxicities and may decrease patient tolerability to S-1. This hypothesis may explain the inferior DFS in patients with low intratumoral DPD expression, compared to patients with high DPD expression, when receiving adjuvant S-1 chemotherapy. Second, high intratumoral DPD *per se* may confer more sensitivity to S-1 than low intratumoral DPD in GC patients. Shimizu et al. reported that the response rate was significantly higher in metastatic gastric scirrhous carcinoma patients with DPD-positive tumors than in those with DPD-negative tumors when treated with S-1-based chemotherapy [29]. All the above suggestions are hypothesis-generating, and more studies are required.

In addition, more aspects need to be further elucidated. The standardization of qRT-PCR methods and the optimization of a cut-off point for DPD mRNA levels in FFPE tumor samples must be conducted. The investigators from the ACTS-GC group used the lowest tertile of intratumoral DPD mRNA levels as a cut-off point [32]. In our study, the lowest quartile was used as a cut-off. When we conducted another analyses using the lowest tertile of intratumoral DPD levels as a cut-off, the impact of DPD expression levels on predicting DFS was still valid in the univariate (S3 Fig) and multivariate analyses (S3 Table). The most appropriate cut-off point of intratumoral DPD mRNA expression levels for predicting treatment outcomes of adjuvant S-1 therapy needs to be validated in future studies. In addition, intratumoral DPD protein expression levels measured by IHC scores (staining intensity multiplied by the percentage of stained area) were also related to different treatment outcomes in our study. However, in the study by the ACTS-GC group, in which the protein expression levels were measured by staining intensity only, the authors could not find any association between IHC results and treatment outcomes [32]. In addition, the ACTS-GC investigators reported that intratumoral TS mRNA expression levels were also predictive of the efficacy of adjuvant S-1 chemotherapy. However, in our study, the expression levels of TS—whether it was measured by IHC or qRT-PCR—were not related to survival outcomes of adjuvant S-1 therapy. Therefore, these different observations between the ACTS-GC investigators and us (the usefulness of intratumoral DPD protein expression levels measured by IHC and TS mRNA expression levels in predicting treatment outcomes) need to be further investigated in the future.

In conclusion, when receiving adjuvant S-1 therapy, GC patients with high intratumoral DPD expression did not have inferior outcome to those with low DPD expression. Instead, low mRNA or protein expression of DPD was related to poor DFS. Administration of lower doses of S-1 due to toxicities might have led to this unexpected inferior treatment outcome in patients with low DPD levels. The expression levels of other fluoropyrimidine-metabolizing enzymes (TS, OPRT, and TP) were not related to survival outcomes of adjuvant S-1 treatment. Future large prospective studies on biomarkers predictive of the efficacy of adjuvant S-1 treatment are warranted.

## Supporting Information

S1 Fig Survival outcomes according to stages: (A) disease-free survival and (B) overall survival. (DOCX)

S2 Fig Distribution of (A) IHC scores of DPD and (B) mRNA expression levels of DPD. (C) The correlation between IHC scores and mRNA expression levels of DPD.
(DOCX)

S3 Fig Disease-free survival curves according to intratumoral mRNA expression levels of DPD (when patients are classified into tertiles). (DOCX)

S1 Table Toxicities developed during S-1 chemotherapy (per patient). (DOCX)

S2 Table The comparison of the delivery of S-1 and developed toxicities between intratumoral DPD-low and DPD-high expression groups [(A) by IHC scores and (B) by mRNA expression levels]. (DOCX)

S3 Table Impact of the intratumoral DPD mRNA expression levels on disease-free survival. The DPD mRNA expression levels were divided into 3 groups (tertiles).
(DOCX)
