# Supplementary material for: Impact of Intratumoral Expression Levels of Fluoropyrimidine-Metabolizing Enzymes on Treatment Outcomes of Adjuvant S-1 Therapy in Gastric Cancer
Source: PLoS One. 2015 Mar 20;10(3):e0120324. doi: 10.1371/journal.pone.0120324 (PMC4368508; doi:10.1371/journal.pone.0120324)
Supplement: S1 Fig — (DOCX) [file pone.0120324.s001.docx]

**S1 Figure.** Survival outcomes according to stages: (A) disease-free survival and (B) overall survival

**(A)**

**(B)**
