# Supplementary material for: Impact of Intratumoral Expression Levels of Fluoropyrimidine-Metabolizing Enzymes on Treatment Outcomes of Adjuvant S-1 Therapy in Gastric Cancer
Source: PLoS One. 2015 Mar 20;10(3):e0120324. doi: 10.1371/journal.pone.0120324 (PMC4368508; doi:10.1371/journal.pone.0120324)
Supplement: S2 Fig — (C) The correlation between IHC scores and mRNA expression levels of DPD. (DOCX) [file pone.0120324.s002.docx]

**S2 Figure.** Distribution of (A) IHC scores of DPD and (B) mRNA expression levels of DPD. (C) The correlation between IHC scores and mRNA expression levels of DPD

**(A)**

**(B)**

**(C)**
