# Supplementary material for: Impact of Intratumoral Expression Levels of Fluoropyrimidine-Metabolizing Enzymes on Treatment Outcomes of Adjuvant S-1 Therapy in Gastric Cancer
Source: PLoS One. 2015 Mar 20;10(3):e0120324. doi: 10.1371/journal.pone.0120324 (PMC4368508; doi:10.1371/journal.pone.0120324)
Supplement: S3 Fig — (DOCX) [file pone.0120324.s003.docx]

**S3 Figure.** Disease-free survival curves according to intratumoral mRNA expression levels of DPD (when patients are classified into tertiles)
