# Supplementary material for: Impact of Intratumoral Expression Levels of Fluoropyrimidine-Metabolizing Enzymes on Treatment Outcomes of Adjuvant S-1 Therapy in Gastric Cancer
Source: PLoS One. 2015 Mar 20;10(3):e0120324. doi: 10.1371/journal.pone.0120324 (PMC4368508; doi:10.1371/journal.pone.0120324)
Supplement: S1 Table — (DOCX) [file pone.0120324.s004.docx]

**S1 Table.** Toxicities developed during S-1 chemotherapy (per patient)

| N = 184 | Grade 1  (N) | Grade 2  (N) | Grade 3  (N) | Grade 4  (N) | All grades  (N) | Grade 3 or 4  (N) |
| --- | --- | --- | --- | --- | --- | --- |
| **Hematological toxicity** | | | | | | |
| Anemia | 110 | 40 | 1 | 0 | 151 | 1 |
| Neutropenia | 52 | 42 | 22 | 2 | 118 | 24 |
| Thrombocytopenia | 23 | 5 | 0 | 0 | 28 | 0 |
| **Nonhematological toxicity** | | | | | | |
| Hyperbilirubinemia | 72 | 13 | 1 | 0 | 86 | 1 |
| AST/ALT elevation | 49 | 3 | 2 | 0 | 54 | 2 |
| Anorexia | 90 | 53 | 7 | 0 | 150 | 7 |
| Nausea | 82 | 23 | 4 | 0 | 109 | 4 |
| Vomiting | 30 | 6 | 2 | 0 | 38 | 2 |
| Stomatitis | 33 | 18 | 2 | 0 | 53 | 2 |
| Diarrhea | 91 | 34 | 13 | 0 | 138 | 13 |
| Abdominal pain | 43 | 24 | 16 | 0 | 83 | 16 |
| Fatigue | 86 | 34 | 2 | 0 | 122 | 2 |
| Hand–foot syndrome | 24 | 11 | 1 | 0 | 36 | 1 |
