# Supplementary material for: Impact of Intratumoral Expression Levels of Fluoropyrimidine-Metabolizing Enzymes on Treatment Outcomes of Adjuvant S-1 Therapy in Gastric Cancer
Source: PLoS One. 2015 Mar 20;10(3):e0120324. doi: 10.1371/journal.pone.0120324 (PMC4368508; doi:10.1371/journal.pone.0120324)
Supplement: S2 Table — (DOCX) [file pone.0120324.s005.docx]

**S2 Table.** The comparison of the delivery of S-1 and developed toxicities between intratumoral DPD-low and DPD-high expression groups [(A) by IHC scores and (B) by mRNA expression levels]

| **(A)** | **DPD IHC score** | | **P-value** |
| --- | --- | --- | --- |
|  | **< 10 (N = 83)** | **≥ 10 (N = 100)** |  |
| Administered cycles of S-1 therapy (mean) | 7.5 cycles | 7.3 cycles | 0.668^*^ |
| RDI of S-1 therapy (median) | 88.1% | 87.3% | 0.643^†^ |
| Number of patients who had maintained RDI ≥ median value (87.8%) during adjuvant chemotherapy (%)^1^ | 42 (50.6%) | 49 (49.0%) | 0.829 |
| Number of patients who had suffered from ≥ grade 3 hematologic toxicities (%) | 11 (13.3%) | 14 (14.0%) | 0.884^‡^ |
| Number of patients who had suffered from ≥ grade 3 non-hematologic toxicities (%) | 18 (21.7%) | 19 (19.0%) | 0.652^‡^ |

| **(B)** | **DPD mRNA expression** | | **P-value** |
| --- | --- | --- | --- |
|  | **Lowest quartile (1^st^)**  **(N = 45)** | **Other quartiles**  **(2^nd^, 3^rd^ and 4^th^)**  **(N = 134)** |  |
| Administered cycles of S-1 therapy (mean) | 7.2 cycles | 7.5 cycles | 0.452^*^ |
| RDI of S-1 therapy (median) | 83.9% | 89.4% | 0.157 ^†^ |
| Number of patients who had maintained RDI ≥ median value (87.8%) during adjuvant chemotherapy (%)^1^ | 18 (40.0%) | 72 (53.7%) | 0.111^‡^ |
| Number of patients who had suffered from ≥ grade 3 hematologic toxicities (%) | 5 (11.1%) | 19 (14.2%) | 0.601 ^‡^ |
| Number of patients who had suffered from ≥ grade 3 non-hematologic toxicities (%) | 13 (28.9%) | 22 (16.4%) | 0.068 ^‡^ |
| ^*^ t-test; ^†^ Mann-Whitney test; ^‡^ χ^2^-test  ^1^ Median value of RDI during adjuvant S-1 treatment period was 87.8%  Abbreviations: IHC, immunohistochemistry; DPD, dihydropyrimidine dehydrogenase; RDI, relative dose intensity | | | |
