# Supplementary material for: Impact of Intratumoral Expression Levels of Fluoropyrimidine-Metabolizing Enzymes on Treatment Outcomes of Adjuvant S-1 Therapy in Gastric Cancer
Source: PLoS One. 2015 Mar 20;10(3):e0120324. doi: 10.1371/journal.pone.0120324 (PMC4368508; doi:10.1371/journal.pone.0120324)
Supplement: S3 Table — The DPD mRNA expression levels were divided into 3 groups (tertiles). (DOCX) [file pone.0120324.s006.docx]

**S3 Table.** Impact of the intratumoral DPD mRNA expression levels on disease-free survival. The DPD mRNA expression levels were divided into 3 groups (tertiles).

| **mRNA expression (N = 179)** | **Hazard Ratio** | **95% Confidence Interval** | **P-value** |
| --- | --- | --- | --- |
| **Age** |  |  | 0.007 |
| < 60 years | 1.00 | - | - |
| 60–69 years | 0.54 | 0.18–1.69 | 0.293 |
| ≥ 70 years | 2.75 | 1.22–6.20 | 0.015 |
| **Stage** |  |  |  |
| IB/II | 1.00 | - | - |
| III | 3.14 | 1.32–7.47 | 0.009 |
| **mRNA expression of DPD** |  |  |  |
| 2^nd^ to 3^rd^ tertiles | 1.00 | - | - |
| 1^st^ tertile (lowest quartile) | 3.02 | 1.40-6.49 | 0.005 |
| Clinical variables that had P < 0.10 in univariate analyses on DFS (age [< 60 years vs. 60–69 years vs. ≥ 70 years), surgical method [laparoscopic vs. open], lymphatic invasion [no vs. yes], venous invasion [no vs. yes], stage [IB/II vs. III]) and mRNA expression levels of DPD gene were included in this multivariate analysis using a Cox proportional hazards model. A backward stepwise conditional regression was used with P = 0.10 as the entry and P = 0.10 as the removal criteria | | | |
